# Burden of influenza A (H1N1)pdm09 infection among tuberculosis patients: a prospective cohort study

**DOI:** 10.1186/s12879-023-08441-3

**Published:** 2023-08-10

**Authors:** Gulshan Umbreen, Abdul Rehman, Muhammad Avais, Chanda Jabeen, Shakera Sadiq, Rubab Maqsood, Hamad Bin Rashid, Saira Afzal, Mamoona Chaudhry

**Affiliations:** 1 Department of Epidemiology & Public Health, University of veterinary and Animal Sciences, Lahore, Pakistan; 2 Department of Veterinary Medicine, University of veterinary and Animal Sciences, Lahore, Pakistan; 3 Department of Veterinary Surgery, University of Veterinary and Animal Sciences, Lahore, Pakistan; 4 Department of Community Medicine, King Edward Medical University, Lahore, Pakistan

**Keywords:** Influenza, Viruses, Tuberculosis, Humans, Incidence, Prospective studies, Cohort studies

## Abstract

**Background:**

Influenza and tuberculosis both cause significant morbidity and mortality worldwide. Therefore, this study aimed to estimate the burden of influenza A (H1N1)pdm09 virus infection among human tuberculosis patients and the general population.

**Methods:**

A prospective cohort study was conducted among a cohort group (TB positive patients) as exposed and a comparison group (general population) as non-exposed. A total of 304 participants were recruited in both groups and followed for a period of 12 weeks. Of the 304 concurrently enrolled individuals, 152 were TB-positive patients (cohort group) and 152 were from the general population (comparison group).To calculate the sample size, the power of study was kept at 80% for detecting a difference at 5% alpha level assuming the 25% prevalence of respiratory viruses in cohort group compared to 12.5% in general population. An oropharyngeal swab was taken from a participant with symptoms of influenza-like illness (ILI). Samples were tested by conventional reverse transcription polymerase chain reaction (RT-PCR) for the detection of influenza A (H1N1)pdm09. All statistical analyses were conducted using R software.

**Results:**

A total of 95 participants developed influenza-like illness (ILI) symptoms. Among these, 64 tested positive for influenza A(H1N1)pdm09, of which 39 were from the exposed group and 25 were from the non-exposed group. During the 12-week period of follow-up, the influenza A (H1N1)pdm09 incidence rate was 20 per 1000 people. The risk of testing positive for influenza A (H1N1)pdm09 was 1.66 times higher in the exposed group compared to the non-exposed group. The cumulative incidence indicated that 25% of the TB cohort and 16% of the comparison group were at risk of getting influenza A (H1N1)pdm09 during the 12 weeks of follow-up.

**Conclusion:**

Participants from the TB cohort had a higher incidence of influenza A (H1N1)pdm09 than the general population suggesting that they should be prioritized for influenza vaccination.

**Supplementary Information:**

The online version contains supplementary material available at 10.1186/s12879-023-08441-3.

## Introduction

Infectious diseases continue to be one of the largest burdens on humankind. Despite the availability of modern medicine, infectious diseases remain the number one cause of mortality. Two of the most common human respiratory infections are those caused by *Mycobacterium tuberculosis* and influenza virus [1]. Regardless of the availability and accessibility of effective treatment protocols, tuberculosis (TB) ranks second in the list of common infectious diseases. Similarly, even with the availability of preventive interventions and vaccines, influenza infections cause significant morbidity and mortality each year around the world [2]. Pakistan ranks 5th amongst countries for burden of TB, with an estimated 510, 000 new cases and approximately 15,000 TB drug resistant cases every year, accounting for 61% of the TB burden in the Eastern Mediterranean Region of World Health Organization (WHO). In 2021, tuberculosis was the 13th most common cause of death and the second leading infectious killer after COVID-19  above HIV/AIDS [3].

Influenza is a serious threat to human health, especially to those in risk groups, including the immunocompromised, elderly, and very young adults [4]. The WHO estimates that influenza-related respiratory illnesses alone cause 3–5 million cases of severe sickness and 290,000-650,000 fatalities worldwide each year [5]. Pakistan has a high burden of infectious diseases due to its favorable climate and population density (the population of Pakistan is 225 million)[6]. Co-morbidity with respiratory viruses, including influenza A, cause a varying degree of morbidity in TB patients compared to the general population [7]. For example, influenza can weaken the innate immune responses to secondary bacterial infections by impairing T-cell immunity [8].

Increased mortality has been seen in TB patients following influenza infections [9, 10]. A sharp decline in TB prevalence was observed after the 1918 Spanish influenza pandemic, possibly due to higher mortality in co-infected patients. Influenza virus pandemics have led to selectively increased mortality among those who suffer with tuberculosis [11]. Individuals with pulmonary tuberculosis (PTB) are at a higher risk of being infected with the influenza virus which may lead to chronic lung disease, immunosuppression and even death. A better understanding of the co-infection of influenza and TB is crucial for policymakers to prioritize the target population for influenza vaccination. Data on the burden of influenza among TB patients is very limited especially from lower and middle income countries. The present study, therefore aimed to estimate the burden of influenza A (H1N1)pdm09 among TB patients with a goal to generate data to improve strategies for reduction of mortality associated with TB.

## Methods

### Study Design

**Fig. 1:**
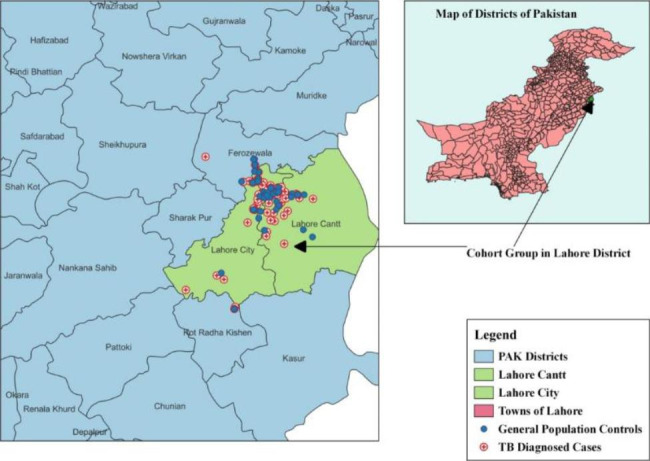
Study area (Lahore) and locations of participants in cohort and comparison group

A prospective cohort study was conducted over a period of 12 weeks to determine the burden of influenza A (H1N1)pdm09 virus infection among TB-positive patients. A comparison group from the general population was included. Lahore is the capital city of Punjab Province and the second-most populous city in Pakistan. It is situated in the northeastern part of the country at 31° 32′ 59′′ latitude and 74° 20′ 37′′ longitude (Fig. 1).

### Study population

#### TB cohort and comparison group

##### Inclusion criteria

An exposed group (TB-positive patients) admitted to a hospital and enrolled in a TB Directly Observed Therapy Program (DOTs) was included. A non-exposed group was selected from individuals who had no history of tuberculosis at the time of the visit, as well as no signs and symptoms of TB, The most common symptom of pulmonary TB is a productive cough lasting more than 2 weeks which may be accompanied by other respiratory symptoms including shortness of breath and chest pain. Individuals were evaluated and excluded if they had any symptoms including fever, fatigue, night sweats, weight loss, and loss of appetite at the time of recruitment.

##### Exclusion criteria

In the exclusion criteria, we excluded all those who had any suspected TB like signs and symptoms. We also excluded all those who had a known history of TB in the past 2 years. We also asked if they had been in close contact with TB patients.

##### Study Procedure

**Fig. 2:**
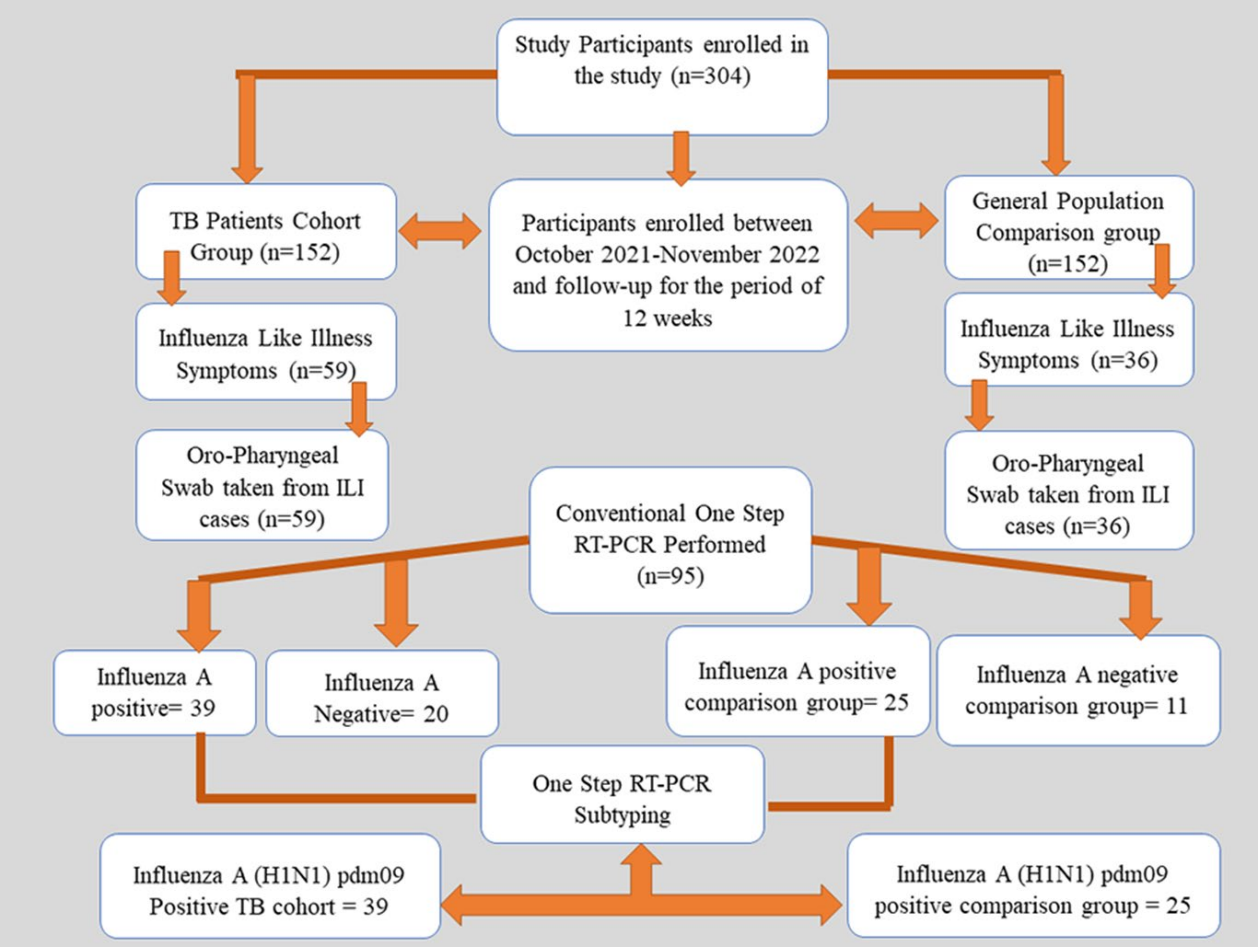
Flow Chart of participants enrolled in the study

Informed consent was obtained from all the participants. Structured questionnaires were used to obtain data on the baseline characteristics of TB patients and adult controls from the general population [7]. The sample size was calculated using winPepi software (version 11.65) with 80% power of detecting a difference, 5% level of significance, assuming the 25% proportion of respiratory viruses in TB patients and 12.5% in general population controls [11]. The calculated sample size was 304 i.e. 152 participants in TB cohort and 152 in comparison group. Participants who fulfilled the inclusion criteria were enrolled in the study and a symptom card was provided for recording symptoms of respiratory illness. The card included information about the date the illness began, body temperature, presence and the severity of symptoms such as fever, headache, muscle aches, cough, and sore throat. We actively contacted each participant every week with phone calls to assess the presence or absence of respiratory illnesses during the study period. An oropharyngeal throat swab was taken from the participant with symptoms of influenza-like illness (ILI). Samples were tested by conventional RT-PCR for influenza A and subtyped for (H1N1)pdm09 (Fig. 2).

**Fig. 3:**
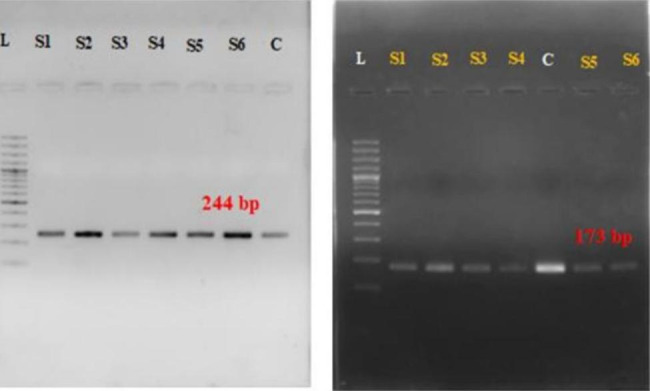
Detection of influenza A (M gene and H1N1pdm09) virus by RT-PCR. (Full-length gels are presented in Supplementary Figure X)

**Laboratory Procedure**: A trained healthcare professional registered with the Pakistan Nursing Council collected oropharyngeal swabs within 3 days of the onset of symptoms. The specimen was collected in a cryovial tube containing 2–3 mL viral transport media and transported under refrigerated conditions to the Disease Surveillance Laboratory, Department of Epidemiology & Public Health, University of Veterinary and Animal Sciences, Lahore, Pakistan. All samples were handled in a class II biosafety cabinet. Viral RNA was extracted using the TRIzol Reagent ® according to the manufacturer’s instructions [12]. Briefly, 250 ul of each sample were used for the extraction of viral RNA. The RNA was eluted with 30 ul of Diethylpyrocarbonate (DEPC) treated water. The eluted RNA was immediately stored at -80 °C for future use. Conventional RT-PCR was performed using a Qiagen one-step RT-PCR kit according to the manufacturer’s recommendations. PCR was conducted with the following cycling conditions: the reverse transcriptase step was carried out at 50 °C for 30 min, 95 °C for 15 min, followed by 35 cycles of amplification [4 °C for 30 s, 55 °C (for M gene)/57°C(for HA gene) for 30 s, 72 °C for 20 s], 72 °C for 7 min then held at 4 °C [13]. PCR products were separated on a 2% agarose gel stained with ethidium bromide. A digital image of the gel was taken using a gel documentation system (Fig. 3).

**Statistical Analysis**: Data sets were entered into the EpiData software (version 3.1, Odense, Denmark, available at http://www.epidata.dk/) validated for errors and inconsistencies by random checking of digital data with the hard copy record, and then exported to Microsoft Excel (version 2016, Microsoft Office, USA) for further processing. All statistical analyses were conducted in R software (version 4.2.1, R Foundation for Statistical Computing, Vienna, Austria) by using ‘epiR and epiDisplay’ packages. Categorical variables were measured in frequencies and proportions. The incidence rate per person per week with a 95% CI was calculated amongst the exposed and non-exposed groups. The cumulative incidence with a 95% confidence interval was calculated to determine the proportion of influenza amongst the TB cohort and the general population. The rate ratio was calculated by dividing the incidence in the exposed group by the incidence in the non-exposed group. If the RR is 1 (or close to 1), it suggests no difference or a little difference in risk (the incidence in each group is the same). An RR > 1 suggests an increased risk of that outcome in the exposed group. An RR < 1 suggests a reduced risk in the exposed group. The location of each cohort was recorded with a smart phone app (Google maps). Maps were created in QGIS version 3.22. biatowieza (available at http://qgis.org/). The shape file of Pakistan boundaries were downloaded from the (https://data.humdata.org/dataset/cod-ab-pak). Using available geographical data, a dot map was produced.

## Results

A total of 304 participants were enrolled in the 12 week prospective cohort study. The majority of participants were female. The TB infected cohort group included 63.1% females and the comparison group had 54.6% females. About 50% of study participants were aged 31–43 years. Almost half of the subjects were married, the rest were single, widowed or divorced. More than 70% lived in urban areas (Table 1).

**Table 1:** Socio-demographic characteristics of respondents (N = 304)

Variables		TB Cohort	Comparison Group
**Gender**	Male	56 (36.84%)	69 (45.39%)
	Female	96 (63.15%)	83 (54.60%)
**Age**	18–30	55 (36.18%)	61 (40.13%)
	31–43	37 (24.34%)	55 (36.18%)
	44–55	18 (11.84%)	21 (13.81%)
	> 55	42 (27.63%)	15 (9.86%)
**Marital Status**	Un-Married	47 (30.92%)	41 (26.97%)
	Married	90 (59.21%)	101 (66.44%)
	Widow	10 (6.57%)	05 (3.28%)
	Divorced	5 (3.28%)	05 (3.28%)
**Education**	Illiterate	81 (53.28%)	42 (27.63%)
	Primary	28(18.42%)	33 (21.71%)
	Secondary	33(21.71%)	24 (15.78%)
	Intermediate	6(3.94%)	17 (11.18%)
	Graduation/ Post-Graduation	4(2.63%)	36 (23.68%)
**Occupation**	Employed	52 (34.21%)	61 (40.13%)
	Un-Employed	68 (44.73%)	39 (25.65%)
	Housewife	29 (19.07%)	40 (26.31%)
	Health Professional	03 (1.97%)	12 (7.89%)
**Residence**	Rural	39 (25.65%)	21 (13.81%)
	Urban	112 (73.68%)	132 (86.84%)
**Monthly Income**	< 15,000	77 (50.65%)	61 (40.13%)
	15,000–30,000	64 (42.10%)	45 (29.60%)
	31,000–45,000	6 (3.94%)	15 (9.86%)
	> 45,000	4 (2.63%)	31(20.39%)
**Family Type**	Nuclear Family	99 (65.13%)	101 (66.44%)
	Extended Family	52 (34.21%)	51 (33.55%)

### Burden of influenza A (H1N1)pdm09 in TB cohort and comparison group

**Fig. 4:**
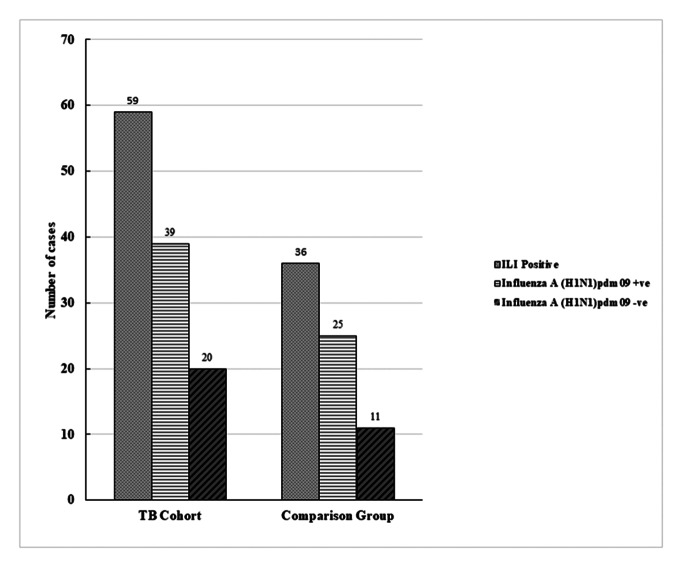
Number of participants positive for ILI and influenza A (H1N1)pdm09 and negative for influenza A (H1N1)pdm09 among TB cohort and comparison group

ILI was found in 39% (59/152) of the TB cohort (95% CI: 31.44–46.75) and 24% (36/152) of the comparison group (95% CI: 17.63–31.04). 66% (39/59) of the TB cohort (95% CI: 53.37–76.86) and 69% (25/36) of the comparison group (95% CI: 53.14–82.99) were positive for A(H1N1)pdm09 (Fig. 4).

#### Incidence rate (IR) in study population

**Fig. 5:**
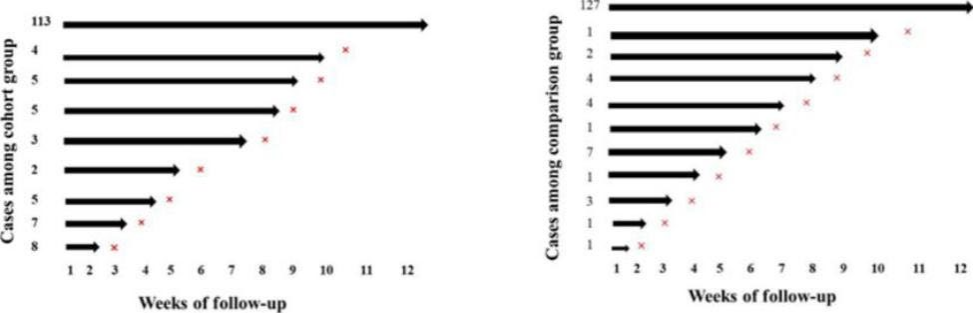
Number of participants in TB cohort and comparison group (general population) enrolled and followed-up for the period of 12 weeks. → represent healthy weeks and × represent disease weeks

A total of 152 individuals each in both the TB cohort and comparison group were enrolled and followed up on for 12 weeks. During this period, 39 people developed influenza, while the rest of 113 individuals remained healthy for the entire 12 weeks. Amongst the comparison group, 25 people developed influenza, while the other 127 remained healthy throughout the 12-week study (Fig. 5). The incidence rate was 25.5 per 1000 person per 12 week in the cohort group and 15.2 per 1000 person per 12 week in comparison group.

### Risk of Influenza A (H1N1)pdm09 in the Study Population

The rate ratio (RR) of influenza A (H1N1)pdm09 was 1.66 (95% CI of 0.99–2.89) in the TB cohort indicating that the risk of influenza A (H1N1)pdm09 was 1.66 times higher in this group compared to the general population.

### Cumulative incidence (CI) among TB Cohort & Comparison Group

The cumulative incidence indicated that 25% (CI: 18.9–33.4) of the TB cohort and 16% (CI: 10.9 − 23.3) of the comparison group (general population) was at risk of getting influenza during the 12-week follow-up period (Table 2).

**Table 2:** Cumulative Incidence of influenza A (H1N1)pdm09 among the TB cohort and comparison group during the 12 week of follow-up

	1 Week	2 Week	3 Week	4 Week	5 Week	6 Week	7 Week	8 Week	9 Week	10 Week	11 Week	12 Week
New Cases among TB Cohort	0	0	8	7	5	2	0	3	5	5	4	0
Cumulative Incidence	0	0	8	15	20	22	22	25	30	35	39	39
New Cases among comparison group	0	1	1	3	1	7	1	4	4	2	0	1
Cumulative Incidence	0	1	2	5	6	13	14	18	22	24	24	25

### Positivity % of Influenza A (H1N1)pdm09 among TB cohort and comparison group

**Fig. 6:**
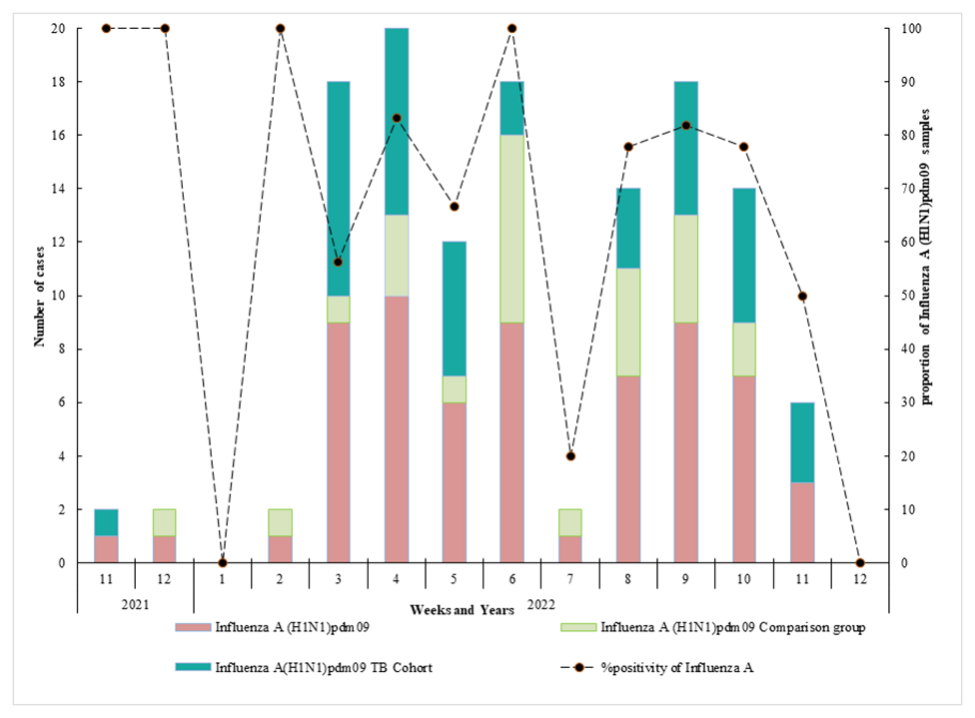
Number and percentage positivity of influenza A(H1N1)pdm09 positive samples among TB cohort and comparison group by duration of study

Among all ILI cases (n = 95), 67% were A(H1N1)pdm09 positive. Out of these 67% (n = 64), 61% were from the TB cohort and 39% from the control group (Fig. 6). The highest proportion of A(H1N1)pdm09 detections was recorded during the 6th week of the follow-up period.

### Laboratory analysis

## Discussion

Influenza and tuberculosis both cause significant morbidity and mortality worldwide [14]. Individuals with pulmonary tuberculosis (PTB) are more likely to get a severe influenza virus infection, which can cause chronic lung disease, immunosuppression, and death [15]. A better understanding of the co-infection of influenza and TB is crucial for policymakers to prioritize the target population for influenza vaccination [16]. Data on the burden of influenza among TB patients is very limited. Therefore, this study aims to estimate the burden of the influenza A (H1N1)pdm09 virus infection amongst TB patients compared to the general population.

In the current study, the proportion of influenza A (H1N1)pdm09 positive cases were higher in males compared to females in the TB cohort. Similarly, in the comparison group, men had more influenza A (H1N1)pdm09 positive cases than women whilst the drivers of this are not known, men in Pakistan usually participate in more outdoor activities and are at a higher risk of exposure to diseases like influenza through close interaction and contact with people who are sick, or contaminated objects or surfaces [17]. Women have also been shown to have a higher compliance with hygienic practices such as hand hygiene/washing, which may have decreased the likelihood of viral infections [18]. Many studies have shown that females have a greater adaptive immune system than males [19–22]. In general, females have higher levels of innate immune cell activity than males, especially at reproductive ages. These cells include dendritic cells (DCs) and macrophages, as well as the overall general inflammatory response [21, 23]. Furthermore, females have greater T-cell counts i.e. CD3 + and CD4+, as well as a higher CD4 + and CD8 + ratio, than males, whereas males have higher frequencies of CD8 + T cells and NK cells [24]. On the other hand, at the time of an influenza A virus infection, testosterone reduces inflammatory monocyte infiltration and pulmonary inflammation [25]. These all provide some mechanistic possibilities for the findings of the current study.

Influenza viruses can cause infection in individuals of any age. However, age groupings can have an impact on the disease’s epidemiology [26, 27]. In our study, adults in the age category 18–43 had the highest percentage of influenza A (H1N1)pdm09 positivity in both groups i.e. TB cohort and comparison group. Previously, a higher positive proportion of influenza A cases were detected in adults ranging from 16 to 30 years of age in a sentinel surveillance of ILI and SARI patients in Lahore, Pakistan [28]. Through significant physiological and behavioral changes that take place throughout life, age may potentially have a varied impact on the outcome of influenza virus infection in males and females [29]. In the present study, the incidence of influenza A virus was higher in the TB cohort in comparison to the general population. Similarly previous studies have reported a higher proportion of influenza A virus in TB infected individuals in comparison to the control group [30]. Variations in these estimates could be due to different study designs and/or the characteristics of the population studied.

We observed an incidence rate higher in our TB cohort group in comparison to the general population. Similarly earlier studies have also reported a lower incidence rate amongst the general population [31], [32]. The WHO predicted that the pandemic virus would continue to circulate at the same time as seasonal viruses and could lead to outbreaks [33]. Influenza virus pandemics have led to selectively increased mortality amongst those who suffer with tuberculosis [9, 10] A sharp decline in TB prevalence was observed after the 1918 Spanish influenza pandemic, possibly due to higher mortality in co-infected patients compared to the general population [11]. People at risk have a significantly higher chance of developing severe influenza and influenza complications [34], often due to suppression of their immune systems which can reduce the ability of the body to control infections. Furthermore, this co-infection could deteriorate the underlying illness, increasing the risk of hospitalization and death [35, 36].

Of our enrolled individuals, only 1.32% of the TB cohort and 3.95% of the comparison group received an influenza vaccination in the previous year. This uptake of the influenza vaccine was lower than reported in the non-immunocompromised (59%) and immunocompromised individuals (25%) in one study in the United States [37]. Vaccination remains the most important and effective primary prevention strategy for reducing the influenza burden in both immunocompromised and healthy populations [37]. Our study has two main limitations. Firstly, it was restricted to the influenza A virus only, and other respiratory pathogens i.e. bacteria and viruses were not studied. Secondly, Latent Tuberculosis was not detected in the general population due to financial constraints.

Influenza poses a serious threat to public health, and immunocompromised patients constitute a vulnerable and high-risk population. More research must be done to develop prevention methods to reduce the burden of influenza virus infection in immunocompromised patients including those with TB. The data generated through the current study should be utilized by health authorities to prioritize influenza vaccination of TB infected individuals as part of a strategy to reduce the burden of excess mortality in this vulnerable population.

## Conclusion

TB infected individuals had a higher incidence of influenza A (H1N1)pdm09 than the general population. Infection with the influenza virus is a serious health risk, especially for certain risk groups, including people with TB. These groups should be targeted for annual influenza vaccination.

### Electronic supplementary material

Below is the link to the electronic supplementary material.

Supplementary Material 1

## Data Availability

All data generated in the current study are available from the corresponding author on reasonable request.
