# Supplementary material for: Burden of influenza A (H1N1)pdm09 infection among tuberculosis patients: a prospective cohort study
Source: BMC Infect Dis. 2023 Aug 10;23:526. doi: 10.1186/s12879-023-08441-3 (PMC10413717; doi:10.1186/s12879-023-08441-3)
Supplement: Supplementary file 1 — Supplementary Material 1 [file 12879_2023_8441_MOESM1_ESM.docx]

**Laboratory Analysis:** Detection of influenza A (M gene) virus at 244 bp by RT-PCR.
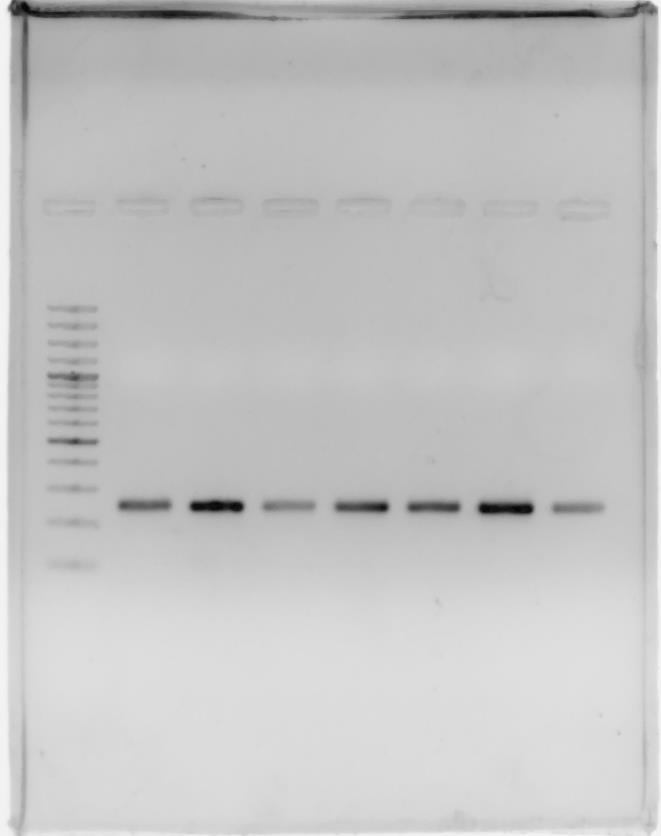


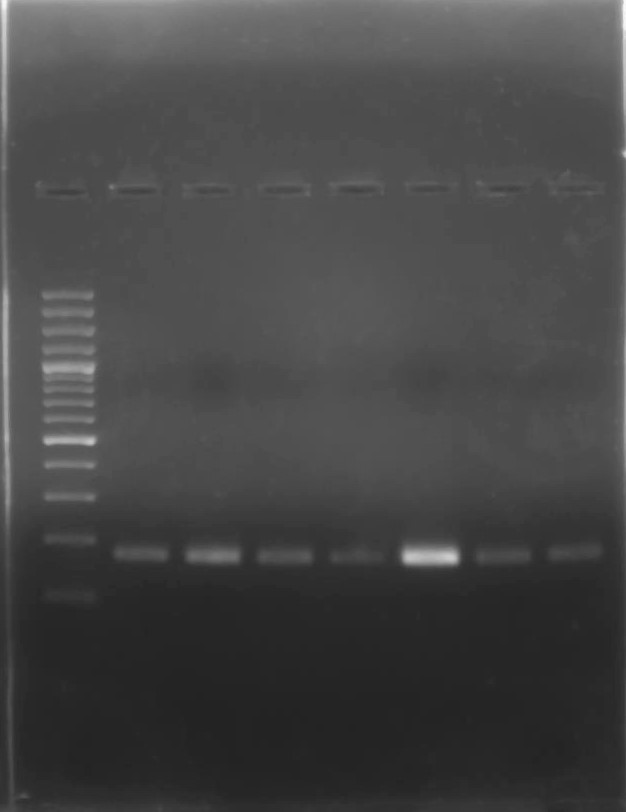


Detection of influenza A (H1N1)pdm09 virus at 173 bp by RT-PCR.
